# Response to Correspondence Regarding “Effects of Autologous Blood‐Derived Extracellular Vesicles on Skin Regeneration and Anti‐Aging: A Clinical Study”

**DOI:** 10.1111/jocd.71019

**Published:** 2026-07-12

**Authors:** Hwansu Kang, Ji‐Hwan Kim, Youin Cho, Dam Go, Jihyun Park, Hyung‐Gi Kim, Sanghoon Park

**Affiliations:** ^1^ Advanced Regenerative Medicine Center ID Hospital Seoul Korea; ^2^ Department of Plastic Surgery ID Hospital Seoul Korea; ^3^ Department of Chemistry University of Michigan Ann Arbor Michigan USA

Dear Editor,

The authors sincerely thank the correspondents for their thoughtful engagement with the article. The authors welcome the opportunity to address the concerns raised and to clarify the methodological and interpretive basis of the findings.

Regarding the timeline of clinical improvement, the correspondents suggested that early outcomes might be attributable to post‐injection edema. While it is acknowledged that canonical dermal remodeling typically unfolds over weeks, the observations extended well beyond the five‐day assessment. All clinical parameters, including wrinkle depth, lifting, and skin density measured via 3D imaging and Cutometer MPA 580, exhibited progressive and statistically significant improvements from day five through the three‐week follow‐up. The sustained enhancement in skin density and elasticity at three weeks points to a biological response that transcends transient edema. To more rigorously establish long‐term durability, the planned follow‐up protocol will extend the observation period to six months.

Regarding study design, the authors fully acknowledge the inherent limitations of a single‐arm, exploratory investigation. The absence of a vehicle control group limits the ability to isolate EV‐specific effects from non‐specific tissue responses. To address this directly, the authors are designing a randomized, split‐face controlled trial as the next phase of research. This study will incorporate the validated Cutometer MPA 580 methodology alongside high‐frequency ultrasound for longitudinal dermal density mapping, and will include a six‐month observation window to provide more rigorous comparative data.

Regarding extracellular vesicle isolation and characterization, size‐exclusion chromatography (SEC) was selected based on its reproducibility and established effectiveness in removing the majority of soluble plasma proteins. Consistent with MISEV2023 guidelines, it is acknowledged that certain lipoproteins may co‐isolate with EVs. To ensure higher isolate purity and mechanistic clarity, future characterization protocols will incorporate lipoprotein‐associated markers (e.g., ApoB/ApoA1) and systematic reporting of EV‐to‐protein ratios.

In conclusion, the findings offer preliminary clinical evidence supporting the potential of autologous blood‐derived EVs in aesthetic medicine. In response to the concerns raised, the authors have outlined a series of targeted improvements—including a randomized controlled design, extended follow‐up, high‐frequency ultrasound endpoints, and enhanced EV characterization—that will form the basis of the next investigation. The authors remain committed to advancing the scientific rigor of this field and welcome continued scholarly dialogue.

## Consent

No written consent has been obtained from the patients as there is no patient‐identifiable data included.

## Conflicts of Interest

The authors declare no conflicts of interest.

## Data Availability

The data that support the findings of this study are available from the corresponding author upon reasonable request.

